# Association of Platelet Trajectory Patterns with In-Hospital Mortality in Critically III Adults with Acute Kidney Injury Receiving Continuous Renal Replacement Therapy

**DOI:** 10.21203/rs.3.rs-6502534/v1

**Published:** 2025-06-03

**Authors:** Tomonori Takeuchi, Udeme Ekrikpo, Joshua Lambert, Nathan Clay, Diego Sanchez Hernandez, Lan Bui, Chloe Braun, Stefania Renzi, Kianoush Kashani, Ashita Tolwani, Javier Neyra

**Affiliations:** University of Alabama at Birmingham; University of Alabama at Birmingham; University of Cincinnati College of Nursing; University of Illinois College of Medicine; Instituto Nacional de Cardiologia Ignacio Chavez; Lloyd L. Gregory School of Pharmacy, Palm Beach Atlantic University; University of Alabama at Birmingham; University of Alabama at Birmingham; Mayo Clinic; University of Alabama at Birmingham; University of Alabama at Birmingham

**Keywords:** Platelet, AKI, CRRT, mortality

## Abstract

**Background:**

Thrombocytopenia commonly occurs in critically ill patients with acute kidney injury (AKI) receiving continuous renal replacement therapy (CRRT) and is associated with poor outcomes. While previous studies have examined static platelet counts and relative declines, the prognostic significance of platelet trajectory patterns before and after CRRT initiation remains unclear. This study investigates the association between platelet count trajectories and in-hospital mortality in critically ill adult patients with AKI receiving CRRT.

**Methods:**

This study is a single-center retrospective cohort study utilizing electronic health record (EHR) data from critically ill adults (≥18 years) with AKI who received CRRT at the University of Alabama at Birmingham between January 2012 and December 2023. Platelet trajectories were assessed using mixed-effects models incorporating all platelet measurements within seven days before and after CRRT initiation. The slopes of platelet count change were categorized as descending (≤ −10 × 10^3^/μL/day), stable (−10 to 10), or ascending (≥ 10), creating a total of nine trajectory patterns by combining the three pre-CRRT and three during-CRRT categories. The primary outcome was in-hospital mortality, which was analyzed in relation to these trajectory patterns using logistic regression models adjusted for demographics, comorbidities, Sequential Organ Failure Assessment (SOFA) score, medications, and use of other organ support devices as covariates.

**Results:**

The study included 2,226 critically ill adults with AKI on CRRT, encompassing 8,569 patient-days before and 11,699 patient-days after CRRT initiation for platelet slope calculation. The median age was 59 years (IQR 48–69) and the median SOFA score at CRRT initiation was 12 (9–14). The in-hospital mortality rate was 57.8%. Before CRRT initiation, the most common trajectory was descending (46.2%), while after CRRT initiation, the stable pattern was most prevalent (58.3%). All patterns with a descending platelet trajectory during-CRRT were significantly associated with higher in-hospital mortality (aOR 1.92 [95% CI: 1.16–3.21] for ascending-descending; 1.49 [1.07–2.08] for stable-descending; 1.52 [1.04–2.23] for descending-descending). In contrast, all patterns with stable or increasing platelet counts during-CRRT tended to have lower mortality.

**Conclusions:**

A descending platelet trajectory after CRRT initiation was independently associated with increased in-hospital mortality.

## Introduction

Critically ill patients frequently develop acute kidney injury (AKI). When solute removal and precise fluid management are required, continuous renal replacement therapy (CRRT) is often utilized, taking into account the severity of illness and hemodynamic status of the recipient. It is well established that patients requiring CRRT have poor clinical outcomes, with reported in-hospital mortality exceeding 50% (1, 2).

Thrombocytopenia commonly occurs in critically ill patients and is associated with poor clinical outcomes. An international cohort study reported that approximately 43% of intensive care unit (ICU) patients developed thrombocytopenia, which was linked to an increased risk of bleeding and mortality(3). Similarly, patients who initiate CRRT with thrombocytopenia have significantly higher mortality compared to those with normal platelet counts(4). Given these findings, platelet count can be considered a relevant clinical parameter in ICU patients undergoing CRRT. In the ICU, platelet count reductions occur regardless of CRRT initiation due to underlying conditions such as systemic inflammation and sepsis(5). Additionally, CRRT itself can contribute to thrombocytopenia through mechanisms including hemodilution, circuit clotting, and platelets consumption or destruction (6, 7). These multifactorial clinical conditions cause platelet counts to fluctuate dynamically before and after CRRT initiation.

Despite this, most previous studies evaluating platelet count in CRRT patients have treated it as a static parameter without considering its dynamic trajectory around CRRT initiation. Therefore, we aimed to investigate the relationship of platelet count trajectories before and after CRRT initiation with in-hospital mortality, providing insights into the prognostic significance of platelet dynamics in critically ill patients with AKI receiving CRRT.

## Methods

### Study Design and Data Sources

This is a single-center retrospective cohort study utilizing electronic health records (EHR) data from the University of Alabama at Birmingham (UAB), an academic medical center in the United States. Extracted variables included sociodemographic characteristics, comorbidities, parameters relevant to Sequential Organ Failure Assessment (SOFA) score(8), organ support device utilization, medications, and laboratory data. The study received approval from the UAB Institutional Review Board (IRB-300009692). The dataset used was completely de-identified and, being retrospective, did not require informed consent.

### Study Participants

**Figure 1:**
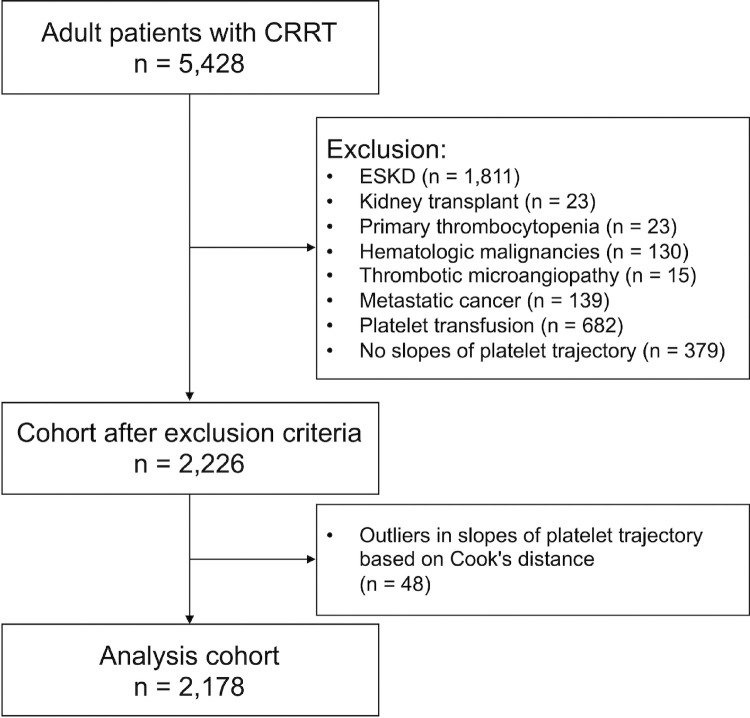
Study Flow Diagram Abbreviation: CRRT, continuous renal replacement therapy; ESKD, end-stage kidney disease.

Our study included critically ill adults (aged ≥18 years) who received CRRT in the ICU between January 2012 and December 2023. Continuous veno-venous hemodiafiltration (CVVHDF) was the primary CRRT modality used in the study, predominantly with regional citrate anticoagulation and HF 1000 filters. Patients with a history of end-stage kidney disease (ESKD) or kidney transplant were excluded. Patients with conditions such as primary thrombocytopenia, hematologic malignancies, thrombotic microangiopathy (except for disseminated intravascular coagulation), and metastatic cancer were excluded. These conditions were identified based on International Classification of Diseases (ICD) codes (**Supplementary Table S1**). Additionally, patients who received platelet transfusion during hospitalization or had insufficient platelet measurements for slope calculation (fewer than two measurements before and after CRRT initiation) were excluded (Figure 1). An average of 9.4 measurements were used for the pre-CRRT slope calculation, and 7.7 for the during-CRRT slope calculation.

### Primary Outcome and Independent Variable

**Figure 2:**
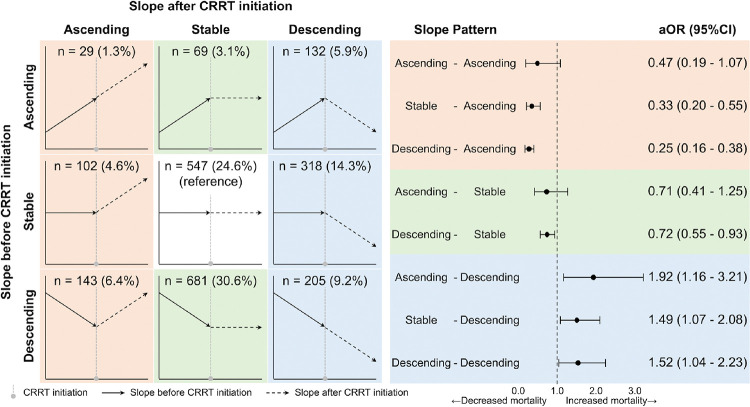
Frequency of Platelet Slope Patterns and Their Association with In-Hospital Mortality Abbreviation: CRRT, continuous renal replacement therapy; aOR, adjusted odds ratio; CI, confidence interval.

The primary outcome was in-hospital mortality. The main independent variable was a combination of the slopes of platelet count before and after CRRT initiation. The slope of the platelet trajectory is defined as a measure that captures the average daily change in platelet count over the specified period and is expressed as 10^3^/μL/day. The slope after CRRT initiation was calculated using all platelet count measurements taken within the first seven days of CRRT initiation, while the slope before CRRT initiation was computed using all measurements from the seven days preceding CRRT initiation. Both slopes (pre-CRRT and during-CRRT) were estimated separately using linear mixed-effects models with platelet count as the dependent variable and days from CRRT initiation (negative for pre-CRRT, positive for during-CRRT) as the independent variable. These models included random intercepts and slopes to capture both the overall cohort trend and individual patient variability. For each patient, individual platelet slopes were calculated by combining the fixed-effect slope estimate with the patient-specific random slope effect. The resulting individual slopes were then categorized into three groups based on cutoff values of −10 and 10: slopes ≤ −10 were classified as “descending,” slopes between −10 and 10 as “stable,” and slopes ≥ 10 as “ascending.” The cutoff values for the slope categories were determined based on the observed distribution of the slope as well as the fact that platelet measurements can have a degree of variability (9) (**Supplemental Figure S1**). Because both pre-CRRT and during-CRRT slopes were categorized separately into these three groups, the combinations resulted in nine distinct categories, including: “Descending-Descending,” “Descending-Stable,” “Descending-Ascending,” “Stable-Descending,” “Stable-Stable,” “Stable-Ascending,” “Ascending-Descending,” “Ascending-Stable,” “Ascending-Ascending” (Figure 2).

Additional independent variables included age, sex, race (Black, White, or other), body weight at CRRT initiation, Charlson Comorbidity Index(9), comorbidies such as liver disease and chronic kidney disease (CKD), baseline serum creatinine (SCr), SOFA score at CRRT initiation, and the platelet count at CRRT initiation. Additionally, sepsis, number of vasopressors, use of inotropes, mechanical ventilation (MV), extracorporeal membrane oxygenation (ECMO), ventricular assist devices (VAD), and heparin exposure were assessed from ICU admission to CRRT initiation. Sepsis was defined according to the Sepsis-3 criteria, requiring the identification of infection based on culture tests and antimicrobial administration, along with an increase in the SOFA score by at least two points during this period(10, 11). The baseline SCr was determined as the preadmission outpatient SCr measurement taken nearest to the admission date, within a window of 7 to 365 days prior. In cases where this was unavailable, preadmission inpatient SCr values meeting the same criteria were utilized. If no such value could be identified, the lowest SCr measurement recorded between 7 days before admission and the end of the index hospitalization was considered. Serum creatinine (SCr) measurements taken from RRT session initiation up to a maximum of 72 hours afterward were excluded from the analysis.

ICU-free days and CRRT-free days were defined as the number of days within the 28 days following CRRT initiation during which the patient did not require ICU care or CRRT, respectively. For patients who died within this 28-day timeframe, the number of free days was recorded as zero. Missing values were observed in body weight (n = 180, 8.1%), SOFA score (n = 17, 0.8%), and Baseline SCr (n = 2, 0.1%). These missing data were addressed using Multivariate Imputation by Chained Equations (MICE) (12).

### Statistical Analysis

Baseline characteristics were stratified and compared by platelet slope pattern after CRRT initiation. The Chi-square test was used to compare categorical variables, while the Mann-Whitney U test was used to compare continuous variables across the platelet slope patterns after CRRT initiation.

Multivariate logistic regression models were used to investigate the association between CRRT slope patterns and in-hospital mortality. The slope pattern “Stable-Stable” was set as the reference category, and odds ratios for in-hospital mortality were calculated for the other eight slope patterns. The model was adjusted for the covariates mentioned above.

As a sub-model, we also analyzed platelet slopes before and during CRRT as continuous variables. Additionally, we tested for interaction effects between the platelet slope before and after CRRT initiation, considering both categorical and continuous variables.

To address outliers and influential points in the platelet slopes, Cook’s distance was calculated using a logistic regression model, with in-hospital mortality as the dependent variable and the slope following CRRT initiation as the independent variable(13). Patients with Cook’s distance values exceeding four times the mean Cook’s distance of the cohort were excluded from the analysis. All statistical analyses were performed using R (Version 4.22, Vienna Austria).

## Results

### Cohort Characteristics

The study included 2,226 critically ill adults with AKI on CRRT with 8,569 patient-days before CRRT initiation and 11,699 patient-days after CRRT initiation considered for platelet slope calculation. The median age of the cohort was 59 (interquartile range [IQR] 48 – 69) years, with 39.0% females, 33.8% black race and 59.9% white race (Table 1). Among included patients, 39.2% had CKD and the median of baseline SCr was 1.20 (IQR 0.90 – 2.00) mg/dL. The median SOFA score was 9 (IQR 6–12) at ICU admission and 12 (IQR 9–14) at CRRT initiation. The median duration of hospitalization was 20 days (IQR 11 – 36 days) and the median duration of ICU stay was 13 days (IQR 7 – 24 days). In-hospital mortality was 57.8%, the median of 28-day ICU-free days was 0 days (IQR 0–12 days), and the median of 28-day CRRT-free days was 0 days (IQR 0–19 days).

### Platelet Slope Patterns

Before CRRT initiation, the descending pattern was the most common, observed in 1,029 patients (46.2%), while after CRRT initiation, the stable pattern was the most frequent, seen in 1,297 patients (58.3%) (Table 1). Patients with a descending platelet slope after CRRT initiation were relatively older, had lower body weight, and had a higher prevalence of liver disease and CKD, particularly in comparison to those with an ascending slope. They were also less likely to have received MV between ICU admission and CRRT initiation, had less exposure to heparin during this period, and their platelet counts were higher at CRRT initiation. This group had a higher in-hospital mortality (62.9%) and shorter 28-day ICU-free days (median 0 [IQR 0–10] days) and CRRT-free days (median 0 [IQR 0–19] days) (Tables 1 and Table 2).

Among the nine pre- and during-CRRT slope combination patterns, the most frequent was Descending-Stable, observed in 681 patients (30.6%), while the least frequent was Ascending-Ascending, found in 29 patients (1.3%) (Figure 2).

### Platelet Slope Patterns and In-Hospital Mortality

In the multivariable model investigating in-hospital mortality, 48 patients were excluded as outliers or influential based on Cook’s distance, leaving 2,178 patients for analysis (Figure 1). Among the nine slope-combination patterns, the three patterns in which the platelet slope was descending after CRRT initiation were associated with increased in-hospital mortality (Figure 2). Specifically, Ascending-Descending had an adjusted odds ratio (aOR) of 1.92 (95% CI: 1.16–3.21), Stable-Descending had an aOR of 1.49 (95% CI: 1.07–2.08), and Descending-Descending had an aOR of 1.52 (95% CI: 1.04–2.23). In contrast, all five patterns in which the platelet slope remained Ascending or Stable after CRRT initiation had negative point estimates of aOR, indicating a trend toward decreased in-hospital mortality. Among these, the Stable-Ascending, Descending-Ascending, and Descending-Stable patterns were significantly associated with lower in-hospital mortality. Results for independent variables other than slope patterns are available in **Supplemental Table S3**.

In the model treating slope as a continuous variable, an increase in the slope after CRRT initiation was significantly associated with decreased in-hospital mortality, with an aOR of 0.96 (95% confidence interval [CI]: 0.95–0.96), while the slope before CRRT initiation did not show a significant association with in-hospital mortality (**Supplemental Table S4**). Of note, the interaction term between the platelet slope before and after CRRT was not significant.

## Discussion

In this study, we examined platelet trajectory patterns before and after CRRT initiation and their association with in-hospital mortality. We found that descending platelet slope patterns after CRRT initiation were significantly associated with increased in-hospital mortality, whereas stable or increasing platelet slope patterns after CRRT initiation were associated with lower in-hospital mortality. These findings emphasize that in critically ill patients with AKI undergoing CRRT, dynamic changes in platelet counts over time convey important prognostic information, rather than just the presence of thrombocytopenia at a single time point.

Our results expand and complement previous studies investigating platelet count and clinical outcomes in CRRT patients. A retrospective single-center study in the United States, which included 541 CRRT patients, demonstrated that both baseline thrombocytopenia before CRRT initiation and platelet decline before and after CRRT initiation were associated with increased ICU mortality(4). Additionally, a multicenter prospective observational study in Brazil involving 274 ICU patients who underwent RRT reported that a 60% decrease in platelet count within the first week was linked to worse outcomes, with a mortality rate of 82.6%(14). Furthermore, a single-center retrospective study in China concluded that a three-day platelet decline, rather than absolute platelet count reduction, was associated with increased mortality(15). These studies focused on one-time-point absolute values and two-time-point differences or relative ratios, with either no assessment of changes or a simplified approach to capturing change. They highlight the evolving recognition that platelets may serve as a dynamic prognostic marker in CRRT patients rather than merely a static parameter.

A major strength of our study is the use of a large sample size, along with detailed EHR data with extensive clinical covariates, allowing for robust model adjustments. Unlike prior studies that relied on single time-point platelet values or simple relative changes, we incorporated both platelet slopes before and after CRRT initiation, considering all available platelet measurements and employing mixed-effects models to account for individual variation as well as overall cohort trend, allowing for a more detailed understanding of how temporal platelet dynamics relate to patient outcomes.

Several plausible pathobiological mechanisms may explain the observed relationship between platelet trajectories and mortality in patients who receive CRRT. In acute critical illness (such as severe AKI and sepsis), systemic inflammation and coagulation disturbances accompanied by endothelial cell injury or macrophage activation syndrome can lead to increased platelet activation and consumption(5, 16, 17). Activated platelets may aggregate and form microthrombi in the microcirculation, contributing to disseminated intravascular coagulation and multi-organ dysfunction, including AKI. In our cohort, platelet trajectories remained a strong prognostic marker, consistent with the hypothesis that progressive platelet decline reflects an underlying inflammatory or coagulopathic state that contributes to worse outcomes(18). From a clinical perspective, when a patient undergoing CRRT exhibits a decrease in platelet count, clinicians should assess underlying conditions (e.g., undiagnosed sepsis, possible disseminated coagulation disorders) and CRRT-related factors (e.g., complement activation, filter clotting, anticoagulation therapy, potential heparin-induced thrombocytopenia) (6, 7). Although this study alone cannot determine specific pathophysiological mechanisms or establish causality or its direction, the above considerations suggest that platelet decline—whether in the context of overall systemic condition or CRRT itself—may serve as a meaningful marker of illness severity. Real-time monitoring of platelet trends, rather than relying on absolute platelet values alone, could serve to identify high-risk individuals with AKI receiving CRRT and guide timely interventions and more personalized medicine.

This study has several limitations. First, as a retrospective observational study, it is subject to confounding and selection biases. In particular, excluding patients who received platelet transfusions—those most likely to show significant platelet changes around CRRT—may have introduced selection bias. Second, our study was conducted at a single center, which may limit its generalizability to institutions with different CRRT protocols, anticoagulation strategies, or filter types. Third, we did not measure platelet function or activation, nor did we have data on inflammatory mediators that may influence platelet quality or quantity.

## Conclusion

This study demonstrates that a descending platelet trajectory after CRRT initiation is associated with increased in-hospital mortality. Assessing platelet trajectory patterns may help refine the phenotypic classification of critically ill patients with AKI on CRRT and could aid in prognostic stratification. Further studies are needed to capture platelet trajectories with more detailed resolution and provide insights into the underlying causes of thrombocytopenia, including inflammation and the direct effects of CRRT/p>

## Figures and Tables

**Table 1.** Cohort Characteristics Stratified by Platelet Slope Pattern after CRRT initiation

	Overall (n = 2226)	Descending (n = 655)	Stable (n = 1297)	Ascending (n = 274)	*p*
**Baseline Characteristics**					
Age (years)	59 [48, 69]	60 [49, 69]	59 [48, 69]	56.50 [43, 67]	0.003
Female sex	869 (39.0)	269 (41.1)	502 (38.7)	98 (35.8)	0.297
Race Black	752 (33.8)	224 (34.2)	410 (31.6)	118 (43.1)	0.004
White	1334 (59.9)	397 (60.6)	797 (61.4)	140 (51.1)	
Other	140 (6.3)	34 (5.2)	90 (6.9)	16 (5.8)	
Body weight at ICU admission (kg)	91.7 [77.0, 111.1]	91.2 [76.7, 111.9]	90.9 [75.9, 109.1]	97.8 [82.1, 116.8]	0.001
Charlson Comorbidity Index	3 [0, 4]	3 [0, 4]	3 [0, 4]	2 [0, 4]	0.001
Diabetes Mellitus	865 (38.9)	264 (40.3)	490 (37.8)	111 (40.5)	0.466
Hypertension	1175 (52.8)	367 (56.0)	652 (50.3)	156 (56.9)	0.019
Cardiovascular disease	1413 (63.5)	417 (63.7)	819 (63.1)	177 (64.6)	0.896
Coronary artery disease	1067 (47.9)	307 (46.9)	617 (47.6)	143 (52.2)	0.308
Liver disease	454 (20.4)	125 (19.1)	310 (23.9)	19 (6.9)	<0.001
Chronic kidney disease	873 (39.2)	269 (41.1)	517 (39.9)	87 (31.8)	0.023
Baseline SCr (mg/dL)	1.20 [0.90, 2.00]	1.20 [0.90, 1.95]	1.30 [0.90, 2.00]	1.20 [0.80, 1.98]	0.154
SOFA score at ICU admission	9 [6, 12]	8 [6, 11]	9 [6, 12]	8 [5, 11]	0.002
SOFA score at CRRT initiation	12 [9, 14]	11 [8, 13]	12 [9, 15]	11 [9, 14]	<0.001
Number of vasopressors at CRRT initiation	1 [0, 2]	1 [1, 3]	1 [0, 3]	1 [0, 2]	<0.001
Use of inotropes at CRRT initiation	527 (23.7)	136 (20.8)	335 (25.8)	56 (20.4)	0.018
Sepsis *	2058 (92.5)	614 (93.7)	1190 (91.8)	254 (92.7)	0.287
MV *	1705 (76.6)	491 (75.0)	985 (75.9)	229 (83.6)	0.013
ECMO *	149 (6.7)	36 (5.5)	103 (7.9)	10 (3.6)	0.012
VAD *	84 (3.8)	15 (2.3)	60 (4.6)	9 (3.3)	0.034
Heparin exposure *	1596 (71.7)	468 (71.5)	908 (70.0)	220 (80.3)	0.003
**Clinical Outcomes**					
In-hospital mortality	1287 (57.8)	412 (62.9)	781 (60.2)	94 (34.3)	<0.001
Hospital length of stay (days)	20 [11, 36]	20 [10, 34.50]	19 [10, 33]	27 [17, 43.75]	<0.001
ICU length of stay (days)	13 [7, 24]	12 [7, 22]	13 [7, 24]	18 [11, 29]	<0.001
28-day ICU-free days	0 [0, 12]	0 [0, 10]	0 [0, 11]	2 [0, 15]	<0.001
Total duration of CRRT (days)	7 [3, 13]	6 [4, 11]	7 [3, 13]	9 [6, 15]	<0.001
28-day CRRT-free days	0 [0, 19]	0 [0, 19]	0 [0, 18]	13.5 [0, 20]	<0.001

Abbreviation: AKI, acute kidney injury; CRRT, continuous renal replacement therapy; ECMO, extracorporeal membrane oxygenation; ICU, intensive care unit; MV, mechanical ventilation; SCr, serum creatinine; SOFA, Sequential Organ Failure Assessment; VAD, ventricular assist device.

Categorical variables are presented as n (%), and numerical variables are presented as median [IQR].

* Determined as exposure between ICU admission and CRRT initiation.

**Table 2.** Platelet Characteristics Stratified by Platelet Slope Pattern after CRRT Initiation

	Overall (n = 2226)	Descending (n = 655)	Stable (n = 1297)	Ascending (n = 274)	*p*
Platelet count at CRRT initiation (10^3^/μL)	158.9 [101.8, 238.7]	235.0 [171.9, 327.5]	129.6 [81.3, 192.7]	153.8 [101.0, 221.4]	<0.001
Highest platelet count after CRRT initiation (10^3^/μL)	176.5 [112.5, 267.4]	232.6 [161.0, 327.2]	134.1 [87.7, 201.1]	287.6 [210.1, 386.4]	<0.001
Lowest platelet count after CRRT initiation (10^3^/μL)	92.3 [45.6, 155.4]	101.0 [51.3, 167.5]	79.9 [38.6, 137.9]	139.7 [84.9, 206.3]	<0.001
Slope of platelet before CRRT initiation (10^3^/μL/day)	−8.8 [−16.5, −0.1]	−4.5 [−12.8, 7.0]	−10.7 [−17.3, −2.4]	−10.4 [−19.6, −1.9]	<0.001
Slope of platelet after CRRT initiation (10^3^/μL/day)	−4.1 [−12.1,2.4]	−18.3 [−26.8, −13.7]	−2.3 [−5.6, 1.7]	18.5 [13.2, 27.1]	<0.001

Abbreviation: CRRT, continuous renal replacement therapy. Numerical variables are presented as median [IQR].

## Data Availability

Data will be shared upon reasonable request to the corresponding author.

## Abbreviations

aOR: Adjusted Odds Ratio
AKI: Acute Kidney Injury
CI: Confidence Interval
CKD: Chronic Kidney Disease
CRRT: Continuous Renal Replacement Therapy
CVVHDF: Continuous Veno-Venous Hemodiafiltration
ECMO: Extracorporeal Membrane Oxygenation
EHR: Electronic Health Record
ESKD: End-Stage Kidney Disease
ICD: International Classification of Diseases
ICU: Intensive Care Unit
IQR: Interquartile Range
IRB: Institutional Review Board
MICE: Multivariate Imputation by Chained Equations
MV: Mechanical Ventilation
RRT: Renal Replacement Therapy
SCr: Serum Creatinine
SOFA: Sequential Organ Failure Assessment
VAD: Ventricular Assist Device

## References

[1] Uchino S, Kellum JA, Bellomo R, Doig GS, Morimatsu H, Morgera S, Acute renal failure in critically ill patients: a multinational, multicenter study. Jama. 2005;294(7):813–8. 16106006 10.1001/jama.294.7.813

[2] Lee HJ, Son YJ. Factors Associated with In-Hospital Mortality after Continuous Renal Replacement Therapy for Critically Ill Patients: A Systematic Review and Meta-Analysis. Int J Environ Res Public Health. 2020;17(23). 10.3390/ijerph17238781 PMC7730748 33256008

[3] Anthon CT, Pene F, Perner A, Azoulay E, Puxty K, Van De Louw A, Thrombocytopenia and platelet transfusions in ICU patients: an international inception cohort study (PLOT-ICU). Intensive Care Med. 2023;49(11):1327–38. 37812225 10.1007/s00134-023-07225-2 PMC10622358

[4] Guru PK, Singh TD, Akhoundi A, Kashani KB. Association of Thrombocytopenia and Mortality in Critically Ill Patients on Continuous Renal Replacement Therapy. Nephron. 2016;133(3):175–82. 27380175 10.1159/000447543

[5] Hui P, Cook DJ, Lim W, Fraser GA, Arnold DM. The frequency and clinical significance of thrombocytopenia complicating critical illness: a systematic review. Chest. 2011;139(2):271–8. 21071526 10.1378/chest.10-2243

[6] Pasko DA, Mottes TA, Mueller BA. Pre dialysis of blood prime in continuous hemodialysis normalizes pH and electrolytes. Pediatr Nephrol. 2003;18(11):1177–83. 14523635 10.1007/s00467-003-1258-2

[7] de Pont AC, Bouman CS, Bakhtiari K, Schaap MC, Nieuwland R, Sturk A, Predilution versus postdilution during continuous venovenous hemofiltration: a comparison of circuit thrombogenesis. ASAIO J. 2006;52(4):416–22. 16883122 10.1097/01.mat.0000227733.03278.5f

[8] Vincent JL, Moreno R, Takala J, Willatts S, De Mendonça A, Bruining H, The SOFA (Sepsis-related Organ Failure Assessment) score to describe organ dysfunction/failure. On behalf of the Working Group on Sepsis-Related Problems of the European Society of Intensive Care Medicine. Intensive Care Med. 1996;22(7):707–10. 8844239 10.1007/BF01709751

[9] Kim H, Hur M, Lee GH, Kim SW, Moon HW, Yun YM. Performance of Platelet Counting in Thrombocytopenic Samples: Comparison between Mindray BC-6800Plus and Sysmex XN-9000. Diagnostics (Basel). 2021;12(1). 10.3390/diagnostics12010068 PMC8775070 35054235

[10] Seymour CW, Liu VX, Iwashyna TJ, Brunkhorst FM, Rea TD, Scherag A, Assessment of Clinical Criteria for Sepsis: For the Third International Consensus Definitions for Sepsis and Septic Shock (Sepsis-3). Jama. 2016;315(8):762–74. 26903335 10.1001/jama.2016.0288 PMC5433435

[11] Singer M, Deutschman CS, Seymour CW, Shankar-Hari M, Annane D, Bauer M, The Third International Consensus Definitions for Sepsis and Septic Shock (Sepsis-3). Jama. 2016;315(8):801–10. 26903338 10.1001/jama.2016.0287 PMC4968574

[12] Buuren Sv, Groothuis-Oudshoorn K. mice: Multivariate Imputation by Chained Equations in R. Journal of Statistical Software. 2011;45(3):67.

[13] Dennis CR. Detection of Influential Observation in Linear Regression. Technometrics. 1977;19(1):4.

[14] Valente C, Soares M, Rocha E, Cardoso L, Maccariello E. The evaluation of sequential platelet counts has prognostic value for acute kidney injury patients requiring dialysis in the intensive care setting. Clinics (Sao Paulo). 2013;68(6):803–8. 23778497 10.6061/clinics/2013(06)13 PMC3674278

[15] Wu B, Gong D, Xu B, He Q, Liu Z, Ji D. Decreased platelet count in patients receiving continuous veno-venous hemofiltration: a single-center retrospective study. PLoS One. 2014;9(5):e97286. 24824815 10.1371/journal.pone.0097286 PMC4019530

[16] Bedet A, Razazi K, Boissier F, Surenaud M, Hue S, Giraudier S, Mechanisms of Thrombocytopenia During Septic Shock: A Multiplex Cluster Analysis of Endogenous Sepsis Mediators. Shock. 2018;49(6):641–8. 29028771 10.1097/SHK.0000000000001015

[17] François B, Trimoreau F, Vignon P, Fixe P, Praloran V, Gastinne H. Thrombocytopenia in the Sepsis Syndrome: Role of Hemophagocytosis and Macrophage Colony-stimulating Factor. The American Journal of Medicine 1997;103(2):7. 10.1016/s0002-9343(97)00136-8 9274894

[18] Claushuis TA, van Vught LA, Scicluna BP, Wiewel MA, Klein Klouwenberg PM, Hoogendijk AJ, Thrombocytopenia is associated with a dysregulated host response in critically ill sepsis patients. Blood, The Journal of the American Society of Hematology. 2016;127(24):3062–72. 10.1182/blood-2015-11-680744 26956172

